# Comparative Genome Analyses of Wild Type- and Quinolone Resistant *Escherichia coli* Indicate Dissemination of QREC in the Norwegian Broiler Breeding Pyramid

**DOI:** 10.3389/fmicb.2020.00938

**Published:** 2020-05-19

**Authors:** Håkon Kaspersen, Eve Zeyl Fiskebeck, Camilla Sekse, Jannice Schau Slettemeås, Anne Margrete Urdahl, Madelaine Norström, Karin Lagesen, Roger Simm

**Affiliations:** ^1^Norwegian Veterinary Institute, Oslo, Norway; ^2^Institute of Oral Biology, University of Oslo, Oslo, Norway

**Keywords:** QREC, wild type, broiler, quinolone, resistance, dissemination

## Abstract

Quinolones are important antimicrobials for both humans and animals, and resistance toward these compounds is a serious threat to public health. In Norway, quinolone resistant *E. coli* (QREC) have been detected at low levels in a high proportion of broiler flocks, even without the use of quinolones in rearing of broilers. Due to the pyramidal structure of broiler breeding, QREC isolates may be disseminated from grandparent animals down through the pyramid. However, quinolone resistance can also develop in wild type *E. coli* through specific chromosomal mutations, and by horizontal acquisition of plasmid-mediated quinolone resistance genes. The goal of this study was to determine whether QREC is disseminated through the broiler breeding pyramid or developed locally at some stage in the broiler production chain. For this purpose, we whole genome sequenced wild type- and QREC isolates from broiler and parent flocks that had been isolated in the Norwegian monitoring program for antimicrobial resistance in feed, food and animals (NORM-VET) between 2006 and 2017, from 22 different production sites. The sequencing data was used for typing of the isolates, phylogenetic analysis and identification of relevant resistance mechanisms. Highly similar QREC isolates were identified within major sequence types from multiple production sites, suggesting dissemination of QREC isolates in the broiler production chain. The occurrence of potential resistance development among the WT *E. coli* was low, indicating that this may be a rare phenomenon in the Norwegian broiler production. The results indicate that the majority of the observed QREC at the bottom of the broiler production pyramid originates from parent or grandparent animals. These results highlight the importance of surveillance at all levels of the broiler production pyramid and of implementation of proper biosecurity measures to control dissemination of QREC.

## Introduction

Quinolones and fluoroquinolones, hereafter collectively referred to as quinolones, are highly prioritized critically important antimicrobials included in the World Health Organization list of essential medicines (World Health Organization [WHO], 2019), and are regarded as last-line antimicrobials in both human and veterinary medicine. Use of quinolones confers a selection pressure that results in enrichment of a resistant subpopulation of bacteria. In line with this, use of quinolones has been linked to increased occurrence of resistant bacteria in both human and veterinary sectors (Teuber, 2001; Terahara and Nishiura, 2019). The presence of quinolone resistant *E. coli* (QREC) in the broiler production chain may have a public health implication, but data on these aspects are limited and the implications are therefore unclear. Quinolone resistance most often develop in bacteria as a result of spontaneous chromosomal mutations in the quinolone resistance determining region (QRDR) of the genes encoding DNA gyrase or topoisomerase IV (Gosling et al., 2012; Hooper and Jacoby, 2015). Resistance can also develop from mutations of regulatory elements resulting in reduced influx or increased efflux of quinolones (Tavío del et al., 1999; Kern et al., 2000) or through acquisition of plasmid mediated quinolone resistance (PMQR) determinants, including *qnr, oqxAB, qepA* or *aac(6′)-Ib-cr* (Gosling et al., 2012; Machuca et al., 2014, 2016; Yamasaki et al., 2015). Additionally, PMQR determinants have been shown to coexist with resistance genes causing resistance toward other antimicrobials, which enables co-selection (Huang et al., 2012; Slettemeås et al., 2019). Quinolone resistance frequently develop in a stepwise fashion, where a single mutation in *gyrA* is often the initial step (Huseby et al., 2017). Additional mutations in either the same gene or other potential quinolone resistance genes, e.g., *parC* or *marR*, can confer increased resistance toward quinolones, but can also be associated with a fitness cost (Marcusson et al., 2009). However, some mutation combinations have been shown to increase both relative fitness and resistance levels, suggesting that resistant mutants may have an advantage whether quinolones are present or not (Marcusson et al., 2009; Huseby et al., 2017).

Quinolone resistance in *E. coli* have been monitored through the Norwegian monitoring program for antimicrobial resistance in feed, food and animals (NORM-VET) since the start in 2000. A selective method for detecting QREC was implemented in 2014 (NORM/NORM-VET, 2014). Using this selective method together with traditional screening for quinolone resistance among commensal *E. coli*, QREC was detected at low levels in a high proportion of samples from broiler flocks. Since quinolones are not used in Norwegian broiler production, this raised the question why QREC is a common finding in the Norwegian broiler population.

The Norwegian broiler production has a pyramidal structure, with the purebred pedigree at the top, breeding animals (parent and grandparent animals) in the middle, and meat-producing broilers at the bottom, as illustrated in Mo et al. (2014). Day-old grandparent animals are imported from Scotland or Germany to Sweden. Eggs from grandparent animals are imported to Norway and hatched to become parent animals, which lay eggs that become broilers. There is no contact between broiler flocks at the bottom of the pyramid. QREC can, as indicated by our previous study (Kaspersen et al., 2020), be introduced to the production pyramid by breeding animals and then be disseminated clonally down the production pyramid. Another possibility is that QREC develop from wild type (WT) *E. coli* at different locations within the production pyramid. Here, WT *E. coli* may either be disseminated from higher in the breeding pyramid to several production sites and subsequently develop resistance, or may develop resistance at a higher level in the pyramid and subsequently disseminate down the pyramid.

In this study, we used comparative genomics to determine whether QREC is disseminated in the broiler breeding pyramid or develops from WT *E. coli*. The aim was to understand if there is an unknown selective pressure in the broiler houses that can, at least partially, explain the observed occurrence of QREC in broilers.

## Materials and Methods

### Study Design and Isolate Selection

*Escherichia coli* from chicken has been susceptibility tested in the NORM-VET program since it started in 2000. Isolation of *E. coli* has in general been done from fecal (2002, 2004, 2006), boot swab (2009, 2011), or cecal (from 2014), samples from broiler chickens on a biannual basis. However, occasionally samples from layer hens and parent flocks have been included in the program. Each flock is only sampled once per year, and only one random *E. coli* isolate has been obtained from each sample.

The isolates used in the present study are a subset of the *E. coli* isolates that have been isolated in the NORM-VET program and have been stored in the biobank of the Norwegian Veterinary Institute. Isolates were included in the present study based on the following criteria: (I) the production site had been sampled at least three times between 2006 and 2017 and (II) at least one QREC and one WT *E. coli* had been isolated from chickens originating from each production site in this time period. This selection resulted in a total of 106 isolates from 22 production sites, comprising 41 QREC and 65 WT *E. coli*, sampled in the years 2006, 2014, 2016 and 2017. Broiler flocks were sampled in 2006, 2014, and 2016 (*n* = 100), whereas in 2017 only parent flocks were sampled (*n* = 6). In total, each production site was represented by four to eight isolates (Table 1). However, it is not known if the isolates were sampled from the same broiler house each time, as some broiler farms have more than one house.

**TABLE 1:** Number of sequence types (ST) per phenotype and location.

	Sequence types		
Location	Quinolone resistant	Wild type	Total
A	ST162	ST10, ST442, ST5825	4
B	ST131, ST349	ST10, ST1286	4
C	ST162	ST355, ST2040, ST9424	4
D	ST355, ST641, ST4994	ST10, ST5375	5
E	ST155	ST10, ST1286, ST5825	4
F	ST117	ST328, ST5825, ST9427	4
G	ST162	ST10 (3)	4
H	ST355	ST10 (2), ST5825	4
I	ST355	ST48, ST189, ST1730, ST5825	5
J	ST349 (2)	ST648, ST1266	4
K	ST349, ST355, ST752 (2)	ST10, ST4537, ST5825 (2)	8
L	ST131*, ST355 (2)*, ST355	ST117*, ST189, ST9425*, ST9426*	8
M	ST349	ST10 (3), ST1266	5
N	ST131, ST349	ST1286, ST5825	4
O	ST10, ST349 (2)	ST756, ST1266, ST2178, ST5375	7
P	ST355 (2)	ST115, ST3107	4
Q	ST131, ST355	ST1594, ST5825	4
R	ST10, ST355	ST10 (2), ST5825	5
S	ST355	ST10 (2), ST191	4
T	ST355, ST602	ST10, ST1056	4
U	ST191, ST355	ST10, ST752, ST1251, ST6726	6
V	ST115, ST162	ST10, ST69, ST602	5

Location: Broiler production site. Numbers in parentheses denote number of isolates for STs with more than one isolate. *Parent flocks.

All isolates had been susceptibility tested by the broth microdilution assay as part of the NORM-VET program, either using panels from VETMIC^TM^ (Dep. Of Antibiotics, National Veterinary Institute, Sweden) in the years 2006–2013 or Sensititre^®^ (TREK Diagnostics, Ltd.) from 2014. The panels contain different antimicrobial agents, and only the compounds represented in both panels were considered. In addition to ciprofloxacin and nalidixic acid, the panels included ampicillin, tetracycline, gentamicin, chloramphenicol, trimethoprim, cefotaxime, and sulfamethoxazole. In this study, isolates with a minimum inhibitory concentration (MIC) value >0.06 mg/L for ciprofloxacin and/or >16 mg/L for nalidixic acid were defined as QREC, according to epidemiological cut-off (ECOFF) values defined by the European Committee on Antimicrobial Susceptibility Testing (EUCAST).^1^ Isolates with MIC below these values are referred to as WT.

### DNA Extraction and Sequencing

Quinolone resistant *E. coli* isolates were plated onto MacConkey agar with ciprofloxacin (0.06 mg/L) to confirm resistance, while WT isolates were plated onto MacConkey agar. Following incubation at 41.5°C for 21 h, bacteria were harvested directly from the agar plates and DNA was extracted with the QIAmp DNA mini kit (QIAGEN), according to the manufacturer’s instructions. The DNA concentration and purity was determined using a Qubit (QIAGEN) and NanoDrop ONE spectrophotometer (Thermo Scientific), respectively. Gel electrophoresis was used to determine the DNA integrity.

A total of 95 isolates were sequenced in this study, using Nextera DNA Flex library preparation (Illumina) followed by sequencing on HiSeq X (Illumina) spiked with PhiX. The remaining 11 isolates were previously sequenced using Nextera XT and HiSeq 2000 (*n* = 4) or HiSeq 2000R (*n* = 3), or Nextera DNA Flex and HiSeq 3000 (*n* = 1) or HiSeq X (*n* = 3). Library preparation and sequencing was done at the Norwegian Sequencing Centre.^2^

### Quality Control of Raw Reads

All fastq files were quality controlled by fastQC^3^ version 0.11.7. Mash (Ondov et al., 2016) version 1.1 was used to identify contaminants in the fastq files, by using a database of all complete bacterial genomes downloaded from RefSeq. Significant contaminants were defined as hits to other bacteria than *E. coli* with an identity value above 0.95. Residual PhiX (accession number NC_001422.1) was removed with bbduk^4^ version 38.20 with a k-mer size of 31, followed by Trimmomatic (Bolger et al., 2014) version 0.38 to trim low-quality nucleotides using the NexteraPE-PE adapter file, a minimum length setting of 36 and a sliding window of 4:15.

### MLST and Resistance Mechanism Identification

Antimicrobial resistance gene identification by assembly (ARIBA) was used for multi-locus sequence typing (MLST), with the scheme hosted by EnteroBase (Wirth et al., 2006). Genomes with novel or uncertain sequence types (STs) were uploaded to EnteroBase for ST assignment.

Mutations in chromosomal genes related to quinolone resistance and plasmid mediated resistance genes were identified with ARIBA using the MEGARes (Lakin et al., 2017) and ResFinder (Zankari et al., 2012) databases, respectively. For the chromosomal genes, only mutations in the QRDR of *gyrA, gyrB, parC*, and *parE* that led to amino acid substitutions in each encoded protein were included. For the plasmid mediated genes, all genes in the ResFinder database were included in the analysis. An R script^5^ was used to filter the results based on flags reported by ARIBA to ensure high quality of the predicted variant or gene.

### Assembly, Annotation, and Pan Genome Analysis

SPAdes (Bankevich et al., 2012) version 3.12.0 was used to assemble the trimmed reads with “coverage cutoff” set to auto in addition to the “careful” setting. To maximize coverage, both the paired and singleton reads from Trimmomatic were used. Assemblies were error corrected with Pilon (Walker et al., 2014) version 1.22 by mapping the trimmed reads back to the assembly with BWA mem^6^ version 0.7.17. Quast (Gurevich et al., 2013) version 4.6.3 was used for assembly evaluation. Prokka (Seemann, 2014) version 1.13 was used for gene annotation, with five complete *E. coli* genomes used as an annotation reference (Supplementary Table 1). Roary (Page et al., 2015) version 3.12.0 was used for pan-genome analysis.

### Phylogenetic Analysis

To investigate the overall phylogenetic relationship between the isolates, a core gene single nucleotide polymorphism (SNP) tree was calculated. First, SNP sites in the core gene alignment from Roary were concatenated with snp-sites (Page et al., 2016) version 2.4.1. The resulting concatenated SNPs were used in IQ-Tree (Nguyen et al., 2015) version 1.6.8 to create a maximum likelihood (ML) tree. The optimal evolutionary model was selected by using ModelFinder plus (Kalyaanamoorthy et al., 2017) in addition to the ascertainment bias correction (Lewis, 2001). Branch supports were generated with UltraFast bootstrap approximation (Hoang et al., 2018). Isolates were defined as closely related based on previously defined thresholds (Kaspersen et al., 2020).

Major clades (*n* > 4) that were represented by either quinolone resistant isolates only, WT isolates only, or both, were further analyzed separately. First, ParSNP (Treangen et al., 2014) version 1.2 was used to align the pilon-corrected assemblies and identify core genome SNPs. The resulting alignment was format converted by using Harvesttools (Treangen et al., 2014) version 1.2. Then, Gubbins (Croucher et al., 2015) version 2.3.2 was used to remove recombinant sites in the multifasta alignment by using RAxML as treebuilder with the GTRGAMMA model. IQTree was subsequently used to calculate a ML tree from the resulting alignment, using the same settings as described above. All phylogenetic trees were visualized in R using ggtree (Yu et al., 2017). STs that contained both WT and QREC isolates were analyzed in regards to genome similarity using ParSNP.

### Data Management

Figures and tables were generated in and data management was done using R version 3.6.2 (R Core Team, 2018).

## Results

### Resistance Patterns and Mechanisms

Depending on the year of sampling, the isolates had previously been tested against one of two different panels of antimicrobials in the NORM-VET program. The resistance pattern of the isolates included in this study was summarized for each of the nine antimicrobials that were included in both panels (Table 2). Overall, a low occurrence of resistance was observed for all tested antimicrobials except against ciprofloxacin and nalidixic acid. All QREC isolates were resistant to ciprofloxacin and nalidixic acid, 12% were resistant to ampicillin and sulfamethoxazole, 10% to trimethoprim, 7% to tetracycline, and 2% to chloramphenicol. Resistance to gentamicin or cefotaxime was not observed. For the WT isolates, resistance toward ampicillin and sulfamethoxazole was observed in 9% of the isolates, 6% were tetracycline resistant, 3% were trimethoprim resistant and 2% were cefotaxime resistant. All WT isolates were susceptible to chloramphenicol and gentamicin.

**TABLE 2:** Overview of percent (%) resistance (top) for quinolone resistant *E. coli* (QREC) and wild-type *E. coli* (WT), and percent occurrence of identified plasmid-mediated resistance genes (bottom).

	QREC (*n* = 41)	WT (*n* = 65)
	
Antimicrobial	Percent (%) resistance	
CIP	100	0
NAL	100	0
AMP	12	9
SMX	12	9
TET	7	6
TMP	10	3
CHL	2	0
CTX	0	2
GEN	0	0

**Genes**	**Percent (%) occurrence**	

*aph3Ib*	9.8	3.1
*aph6Id*	9.8	3.1
*bla*_TEM–__1__B_	9.8	4.6
*sul2*	7.3	4.6
*dfrA5*	4.9	1.5
*tetA*	4.9	4.6
*aadA1*	2.4	3.1
*aadA5*	2.4	0.0
*bla*_CMY–__2_	2.4	1.5
*catA1*	2.4	0.0
*dfrA1*	2.4	1.5
*dfrA17*	2.4	0.0
*sul3*	2.4	0.0
*tetB*	2.4	1.5
*bla*_TEM–__1__A_	0.0	1.5
*bla*_TEM–__220_	0.0	1.5
*dfrA14*	0.0	1.5
*fosA7*	0.0	1.5
*sul1*	0.0	1.5

Amino acid substitutions in the QRDR of GyrA were only observed in QREC isolates (Table 3), all of which had the S83L substitution. Two QREC isolates had an additional D87N substitution in GyrA. No substitutions in the QRDR of GyrB was observed among the QREC isolates. Some QREC had additional amino acid substitutions in ParC or ParE (Table 3). Four WT isolates had substitutions in the QRDR of either GyrB, ParC, or ParE.

**TABLE 3:** Number of isolates with the respective amino acid substitution in GyrA, GyrB, ParC, and ParE per phenotype.

Protein	AA substitution	Quinolone resistant	Wild type
GyrA	D87N	2	0
	S83L	41	0
GyrB	S463A	0	2
ParC	S57T	0	1
	S80I	4	0
ParE	A512T	1	0
	D475E	14	1
	L488M	1	0

PMQR genes were not detected in any of the isolates, but plasmid mediated resistance genes conferring resistance to other antimicrobials were detected (Table 2). The most abundant plasmid mediated resistance genes among the QREC and WT isolates were *aph3Ib* (9.8 and 3.1%), *aph6Id* (9.8 and 3.1%), *bla*_*TEM*__–__1__B_ (9.8 and 4.6%), *sul2* (7.3 and 4.6%), *dfrA5* (4.9 and 1.5%), *tetA* (4.9 and 4.6%), and *aadA1* (2.4 and 3.1%). Overall, the genotype corresponded to the observed phenotype, except for the *aph* and *aadA* genes, since gentamicin resistance was not observed in the isolates. For detailed resistance patterns and presence/absence of resistance genes, see Supplementary Data Sheet 1.

### Sequence Type Diversity and Phylogenetic Analyses

**FIGURE 1:**
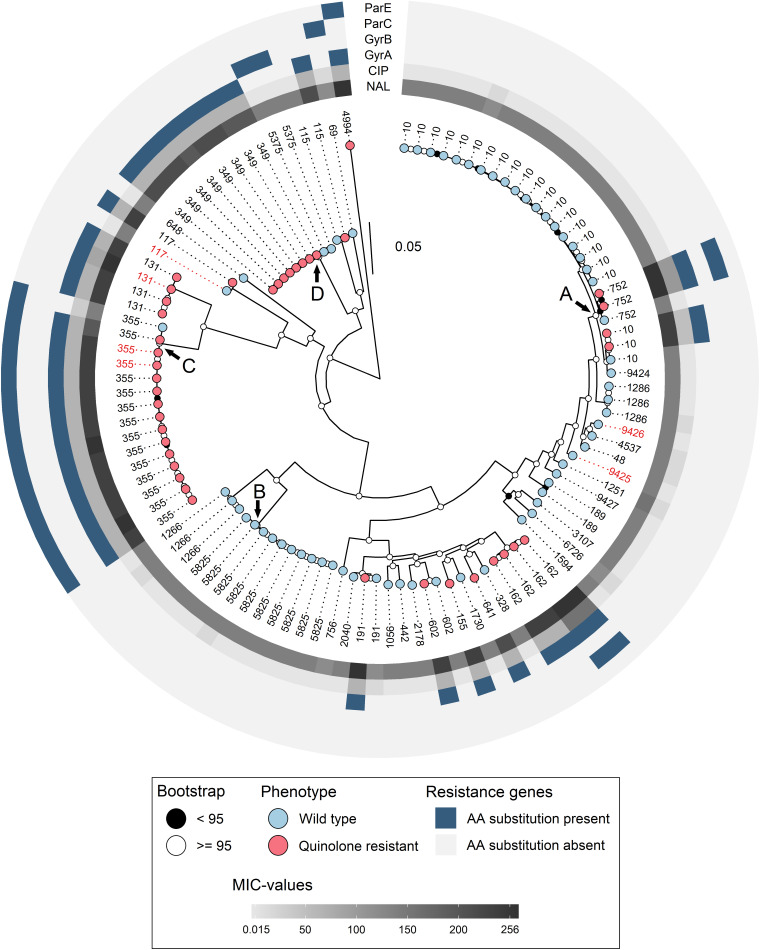
Maximum likelihood SNP tree calculated with IQTree, based on the 2931 core genes identified with Roary. The tree is midpoint rooted for better visualization. Bootstrap values are represented as black (<95) and white (≥ 95) circles on the nodes. Phenotype is represented as blue (wild type) and red (quinolone resistant) on the tip points. Sequence types are denoted as tip labels, red labels represent parent animals. MIC-values for ciprofloxacin (CIP) and nalidixic acid (NAL) are represented as increasing gray color in the innermost circles. Amino acid substitutions in the four genes related to quinolone resistance is denoted as blue in the surrounding circles. Arrows denote clades further investigated. Evolutionary model: GTR + F + ASC + R5.

In total, 37 different STs were detected among the 106 isolates. There were 31 different STs among the 65 WT isolates, and 13 different STs among the 41 QREC isolates (Table 4). Seven different STs contained both quinolone resistant and WT isolates, namely ST752, ST10, ST602, ST191, ST355, ST117, and ST115 (Figure 1). ST10 and ST5825 represented the major STs for WT isolates, while ST349, and ST355 represented the major STs for QREC isolates (Figure 1).

**TABLE 4:** Number of sequence types (ST) per year of isolation and phenotype.

	Sequence types					
Year	Quinolone resistant	*n*	Wild type	*n*	Sum	*n* isolates
2006		0	ST10, ST48, ST69, ST191, ST355, ST756, ST1251, ST1286, ST4537, ST6726	10	10	10
2014	**ST355(12), ST349(8),** ST162(4), ST131(3), **ST10(2),** ST115, ST117, ST155, ST191, ST602, ST4994	11	**ST10(10), ST5825(7)**, ST5375(2), ST115, ST189, ST442, ST602, ST752, ST1056, ST1266, ST1286, ST1594, ST2040, ST2178, ST3107, ST9424, ST9427	17	25	68
2016	ST752(2), ST641	2	**ST10(9), ST5825(3),** ST1266(2), ST189, ST328, ST648, ST1286, ST1730,	8	10	22
2017*	**ST355(2),** ST131	2	ST117, ST9425, ST9426	3	5	6
Total		15		38	50	106

The columns “*n*” denote the number of unique STs for each phenotype per year. The “Sum” column denote the total number of unique STs per year. “*n* isolates” denote the number of isolates per year. Numbers in parentheses denote number of isolates for STs with more than one isolate. Major STs are denoted in bold. *Parent flocks.

The number of isolates and unique STs varied from year to year (Table 4). In 2006 each identified ST only consisted of a single isolate. Four major STs (ST10, ST349, ST355, and ST5825) were identified in 2014 and constituted 57% of the isolates for that year, whereas in 2016, ST10 alone accounted for 41% of the isolates. Both the number of isolates and unique STs were reduced in 2017 when parent flocks were sampled. No ST was overrepresented among these isolates. Only one production site had QREC and WT isolates belonging to the same ST (ST10, Table 1).

**FIGURE 2:**
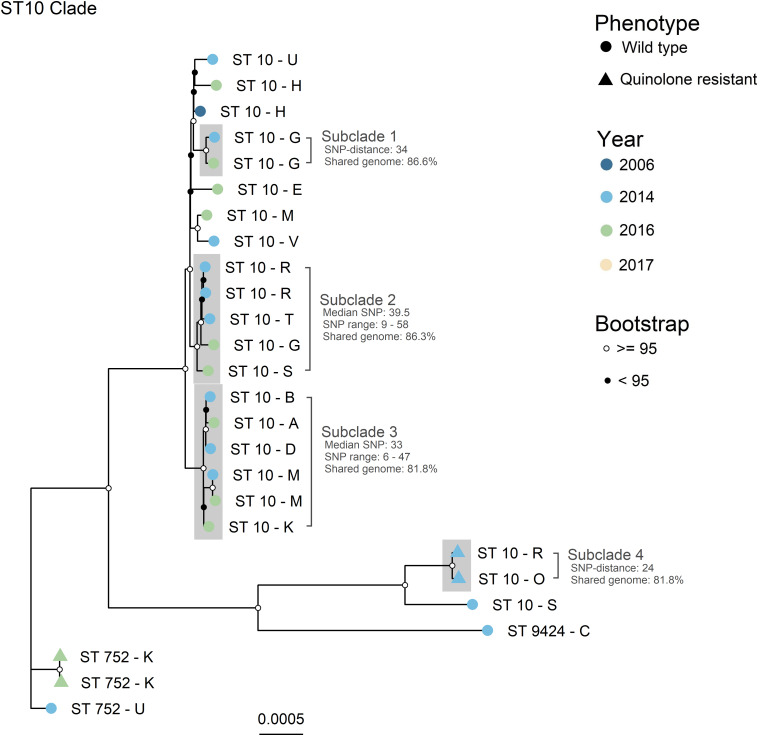
Maximum likelihood core gene SNP tree generated with IQTree for Clade A. Core genome SNPs were identified with ParSNP, and recombinant sites were removed with Gubbins. ST = sequence type. Phenotype is represented by the tip point shapes, and year of isolation represented by the tip point color. Bootstrap values are represented as black and white circles on the internal nodes. Tip labels represent the ST (number) and production site (letter) of each isolate. Subclades of interest are highlighted in gray. Evolutionary model: TVMe + ASC + R2. Total shared genome: 78.3%.

**FIGURE 3:**
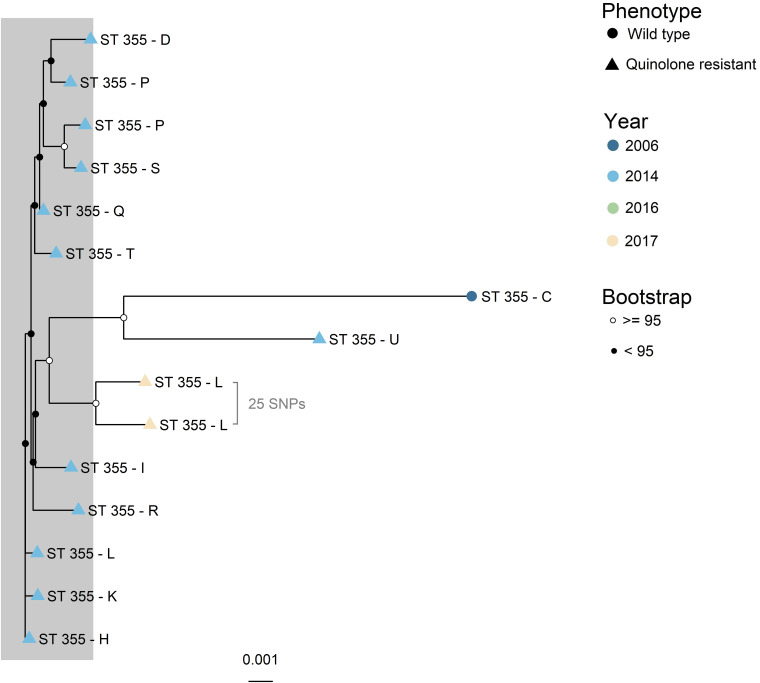
Maximum likelihood core gene SNP tree generated with IQTree for clade C (ST355). Core genome SNPs were identified with ParSNP, and recombinant sites were removed with Gubbins. ST = sequence type. Phenotype is represented by the tip point shapes, and year of isolation represented by the tip point color. Isolates from 2017 were from parent flocks. Bootstrap values are represented as black and white circles on the internal nodes. Similar isolates are marked with a gray box. These had a median SNP distance of 13 and shared 92.5% of their genomes. Evolutionary model: K3P + ASC + R2. Total shared genome: 85.4%.

The four major clades (*n* > 4), illustrated as A – D in Figure 1, were further investigated with higher resolution phylogenetic methods. Clade A (Figure 2) consisted of ST10 (*n* = 22), ST752 (*n* = 3), and ST9424 (*n* = 1) from 15 different production sites. Most of these isolates were isolated in 2014 and 2016, and one in 2006. Most of the ST10 isolates clustered together in the topmost clade, all of which were WT isolates. As demonstrated by subclades 1- 3, phylogenetically related WT isolates were detected from different production sites and years. In addition, two QREC ST10 isolates from the same year but different production sites (Subclade 4 in Figure 2) were seen. Clade C was represented by 15 ST355 isolates from 2014 (*n* = 12), 2017 (*n* = 2) and 2006 (*n* = 1), from 12 different production sites (Figure 3). A majority of the isolates from 2014 (Figure 3, gray box) were separately analyzed in regards to shared genome fraction, and shared 92.5% of their genomes. These isolates had a median SNP distance of 13. The tree topology in clade B (Supplementary Figure 1) and D (Supplementary Figure 2) were judged to be uncertain due to low bootstrap values. Therefore, specific isolates within the trees were not compared, only the tree as a whole. Clade B was represented by ten ST5825 WT isolates from nine different production sites from 2014 (*n* = 7) and 2016 (*n* = 3). These shared 91.7% of their genomes and had a median SNP distance of 18. Finally, clade D was represented by eight QREC isolates of ST349, all isolated in 2014 from four different production sites. Here, a median SNP distance of nine was calculated, and the isolates shared 92.4% of their genomes.

In the seven STs containing both WT and QREC isolates, the two most similar WT and QREC isolates were compared with regards to resistance mechanisms, shared genome, and SNP distances based on the core gene alignment (Table 5). The lowest core gene SNP difference (40 SNPs) was observed between the ST191 isolates, which shared 84.2% of their genomes. Similarly, in ST355 the WT isolate and the closest QREC isolate had a core gene SNP difference of 66, and shared 84.2% of their genomes.

**TABLE 5:** Overview of quinolone resistant *E. coli* and wild type *E. coli* isolate pairs of the same sequence type (ST).

ST	GyrA	ParC	ParE	Year	Clade shared genome (%)	SNP distance	CIP MIC	Location
752	S83L	S80I		2016	83.4^a^	2634^d^	1	K
				2014			0.015	U
10	S83L			2014	80.1^b^	635^e^	0.12	R
				2014			0.015	S
602	S83L			2014	86.4	1795	0.25	T
				2014			0.015	V
191	S83L			2014	84.2	40	0.25	U
				2006			0.03	S
355	S83L		D475E	2014	85.4^c^	66^f^	0.25	Q
			D475E	2006			0.06	C
117	S83L			2014	88.2	2950	0.25	F
				2017*			0.015	L
115	S83L			2014	85.2	397	0.25	V
				2014			0.015	P

Two isolates are included for each ST, where the topmost isolate in each row is quinolone resistant and the bottom one the wild type isolate. Amino acid substitutions are listed in columns “GyrA,” “ParC,” and “ParE.” The shared genome is calculated based on analysis with ParSNP on the two genomes, with results listed as “Clade shared genome (%).” If the clade was comprised of only the wild type and quinolone resistant isolate, this value is based on the shared genome between those two isolates only. The “SNP distance” column represents the SNP distances between the two respective isolates derived from the core gene alignment. CIP MIC = ciprofloxacin minimum inhibitory concentration. Location represents production site. ^*a*^For closest resistant and sensitive isolate: 84.0%. ^*b*^For closest resistant and sensitive isolate: 82.0%. ^*c*^For closest resistant and sensitive isolate: 84.2%. ^*d*^Core genome SNP distance: 104. ^*e*^Core genome SNP distance: 254. ^*f*^Core genome SNP distance: 100. *Parent flock.

## Discussion

This is the first study using phylogenetic methods to compare both QREC and WT isolates from the Norwegian broiler production chain isolated under the auspices of the NORM-VET program. Here, we identified phylogenetically related QREC isolated from geographically distant production sites, indicating vertical dissemination of QREC in the broiler breeding pyramid. Our data also suggest potential rare sporadic development of quinolone resistance in WT isolates at different locations in the broiler production chain. Taken together, our data and the previously reported low-level occurrence of QREC in a high proportion of samples suggest that any unknown selective pressure, if present, is a minor contributor to the total occurrence of QREC observed in the broiler production chain.

In regards to SNP distances, isolates of the same ST from the same production site seemed to be more closely related than isolates of the same ST from different production sites. ST355 and ST349 formed major clades of QREC in the phylogenetic tree in the present study. The relatively high similarity of isolates sampled from different production sites within these two STs suggests that they have a common origin. Occurrence of highly similar QREC ST355 isolates has recently been reported from Iceland and Norway in a study comparing ESBL and QREC isolates from the broiler production chains of Iceland, Norway, and Sweden sampled in 2011–2014 (Myrenås et al., 2018). Furthermore, there were also highly similar QREC isolates of ST349 and ST10 from Sweden and Norway (Myrenås et al., 2018). The present study identified highly similar isolates of WT ST10 in several flocks sampled in 2006, 2014 and 2016, but only two QREC isolates of ST10 and these clustered separately from the majority of the ST10 WT isolates. Since Norway and Iceland both import eggs from Sweden that subsequently become parent animals in the respective countries (Myrenås et al., 2018), this strongly suggests that QREC of ST349 and ST355 have been disseminated by eggs, grandparent or parent animals in this time period. It is noteworthy that QREC of ST349 and ST355 were not detected in samples from the broiler houses in 2016. However, the sample set consisted of only three QREC from this year, and we cannot conclude if this is a trend or sampling bias. Interestingly, while internally related, the highlighted ST355 isolates from 2014 were all phylogenetically distinct from the ST355 isolates from parent animals in 2017.

Findings of ST349 and ST355 QREC isolates in the broiler production environment in several Nordic countries indicate that they are highly successful clones. The quinolone usage among terrestrial livestock in these countries is low (EMA, 2019). This indicates that the presence of the substitutions detected among these isolates may provide a fitness benefit, even in the absence of quinolones. However, this fitness benefit may also be attributed to the QREC lineage itself rather than the specific mutation. All QREC isolates from both STs were found to have the S83L substitution in GyrA, while the ST355 isolates in addition have the D475E substitution in ParE. Isolates with only the S83L substitution have previously been linked with increased fitness (Machuca et al., 2015; Huseby et al., 2017; Wang et al., 2017), which may explain the apparent success of these lineages. The substitutions identified in ParE among the ST355 isolates does not seem to affect the MIC value toward ciprofloxacin and nalidixic acid, as the ST355 and ST349 QREC isolates had the same MIC values.

Wild type *E. coli* and QREC isolates were compared phylogenetically to identify possible development of quinolone resistance among WT *E. coli.* Overall, we regarded the genetic distance between the QREC and WT *E. coli* belonging to the same STs as too high to assume a recent common ancestor, based on previous thresholds (Kaspersen et al., 2020). However, one QREC/WT isolate pair of ST191 had a relatively low genetic distance (40 SNPs) based on the core gene alignment generated with Roary, were isolated 8 years apart, and were from different production sites. Under relatively stable conditions with no apparent selective pressure, *E. coli* have been predicted to develop approximately 80 SNPs over a period of 20 years, given a low rate of horizontal transfer and recombination (Tenaillon et al., 2016). Thus, a difference of 40 SNPs between the ST191 QREC and WT isolates may be expected over 8 years, and indicates phylogenetic relatedness. However, the two isolates only shared 84.2% of their genomes. Horizontal gene transfer and recombination over time may account for this difference. It should be mentioned that the SNP distances mentioned above is based on the alignment of the 2931 core genes. Deeper phylogenetic analysis covering a larger portion of the genomes of the ST191 isolate pair is needed to conclude if these isolates indeed are phylogenetically related. This is evident in the investigated ST355 isolate pair, where 66 SNPs were detected using the core gene alignment described above, while 100 SNPs were detected in the core genome alignment made from the ST355 isolates. It is however unknown if the WT isolate was disseminated before developing resistance, or developed resistance at a higher level in the broiler production pyramid and was subsequently disseminated as QREC down the pyramid. Taken together, the occurrence of resistance development among WT *E. coli* was low in our data. This indicates that such development of resistance is a rare phenomenon in the broiler production environment. As such, our results indicate that *E. coli* are (re)introduced into the broiler houses by dissemination through the breeding pyramid and that some STs can persist in this environment. Given our contention that QREC are mainly disseminated vertically in the broiler breeding pyramid is true, these findings can be confirmed by further investigating QREC from both parent and broiler flocks over time. Wild type isolates with substitutions in GyrB, ParC or ParE were identified. These substitutions have previously been described (Komp Lindgren et al., 2003; Saenz, 2003), and the S463A substitution in GyrB has been identified in *Klebsiella oxytoca* (Lascols et al., 2007). The presence of these substitutions in WT *E. coli* suggest that they alone are not enough to gain a quinolone resistant phenotype. No PMQR determinants were identified in any of the included isolates. This finding is in concordance with previous studies, where a very low occurrence of PMQR were reported (Börjesson et al., 2015; Myrenås et al., 2018), and suggests that PMQR may be a rare finding in the breeding animals that are imported from Scotland or Germany to Sweden. However, some plasmid mediated genes conferring resistance toward cefotaxime, ampicillin, trimethoprim, tetracycline, sulfamethoxazole and chloramphenicol were identified in the present study with low occurrence, mostly in QREC isolates. The presence of these genes in QREC isolates may indicate the possibility of co-selection with the use of other antimicrobial compounds. However, antimicrobial usage in the Norwegian broiler production is very low (NORM/NORM-VET, 2018), and it is unlikely that this is the explanation for the occurrence of QREC in Norwegian broilers. The quinolone usage in the grandparent production in Scotland or Germany is currently unknown. Thus, conclusions based on potential selection of quinolone resistance at the highest levels of the broiler breeding pyramid cannot be drawn.

This study identified major QREC lineages of phylogenetically related isolates across multiple broiler production sites, suggesting vertical dissemination of quinolone resistance in the broiler breeding pyramid. The seemingly low occurrence of quinolone resistance development among WT *E. coli* together with the fact that QREC are found at low levels in a high proportion of samples, suggest that there is no major unknown pressure selecting for quinolone resistant bacteria. Instead, our data indicates that the major contributor to QREC occurrence in the broiler production chain is dissemination of strains originating from parent or grandparent animals. Measures to control occurrence of QREC in broilers should therefore be focused on the higher levels of the broiler breeding pyramid.

## Data Availability Statement

The datasets analyzed for this study can be found in the European Nucleotide Archive, accession numbers PRJEB37581, PRJEB33043, PRJEB33048, and PRJEB36302.

## Author Contributions

AU, MN, KL, RS, CS, JS, and HK conceptualized and designed the study. HK, EF, KL, and RS analyzed the data. EF and KL advised and assisted the phylogenetic analysis. HK, KL, and RS wrote the main body of the manuscript. All authors contributed to manuscript revisions, interpretation of results, and manuscript approval.

## Conflict of Interest

The authors declare that the research was conducted in the absence of any commercial or financial relationships that could be construed as a potential conflict of interest.

## Footnotes

1 www.eucast.org, ECOFFs as of 01.08.2019

2 www.sequencing.uio.no

3 https://www.bioinformatics.babraham.ac.uk/projects/fastqc/, accessed 29.09.2019

4 https://jgi.doe.gov/data-and-tools/bbtools/accessed 29.09.2019

5 https://github.com/hkaspersen/VAMPIR, commitid 54d687a (12^th^ of May)

6 http://bio-bwa.sourceforge.net/, accessed 29.09.2019
