# Supplementary material for: Comparative Genome Analyses of Wild Type- and Quinolone Resistant Escherichia coli Indicate Dissemination of QREC in the Norwegian Broiler Breeding Pyramid
Source: Front Microbiol. 2020 May 19;11:938. doi: 10.3389/fmicb.2020.00938 (PMC7248565; doi:10.3389/fmicb.2020.00938)
Supplement: Supplementary file 2 [file Data_Sheet_2.docx]

Supplementary Material

# Supplementary Data

**Supplementary Data Sheet 1**: Total data on all included isolates (See Excel sheet). Information includes year of isolation, production site of origin, phenotype, minimum inhibitory concentrations values, sequence types, amino acid substitutions in GyrA, GyrB, ParC and ParE, and all detected plasmid mediated resistance genes.

# Supplementary Tables and Figures

## Supplementary Tables

**Supplementary Table 1:** Complete *E. coli* genomes used with Prokka. The table presents the complete reference genomes used when annotating the draft genomes. These were selected as references since they are regarded as highly curated *E. coli* genomes, and were complete assemblies.

| Accession number | Information |
| --- | --- |
| GCF_000005845.2_ASM584v2 | *E. coli* K12 |
| GCF_000008865.2_ASM886v2 | *E. coli* O157:H7 Sakai |
| GCF_000026345.1_ASM2634v1 | *E. coli* IAI39 |
| GCF_000183345.1_ASM18334v1 | *E. coli* O83:H1 NRG 857C |
| GCF_000299455.1_ASM29945v1 | *E. coli* O104:H4 2011C-3493 |

## Supplementary Figures


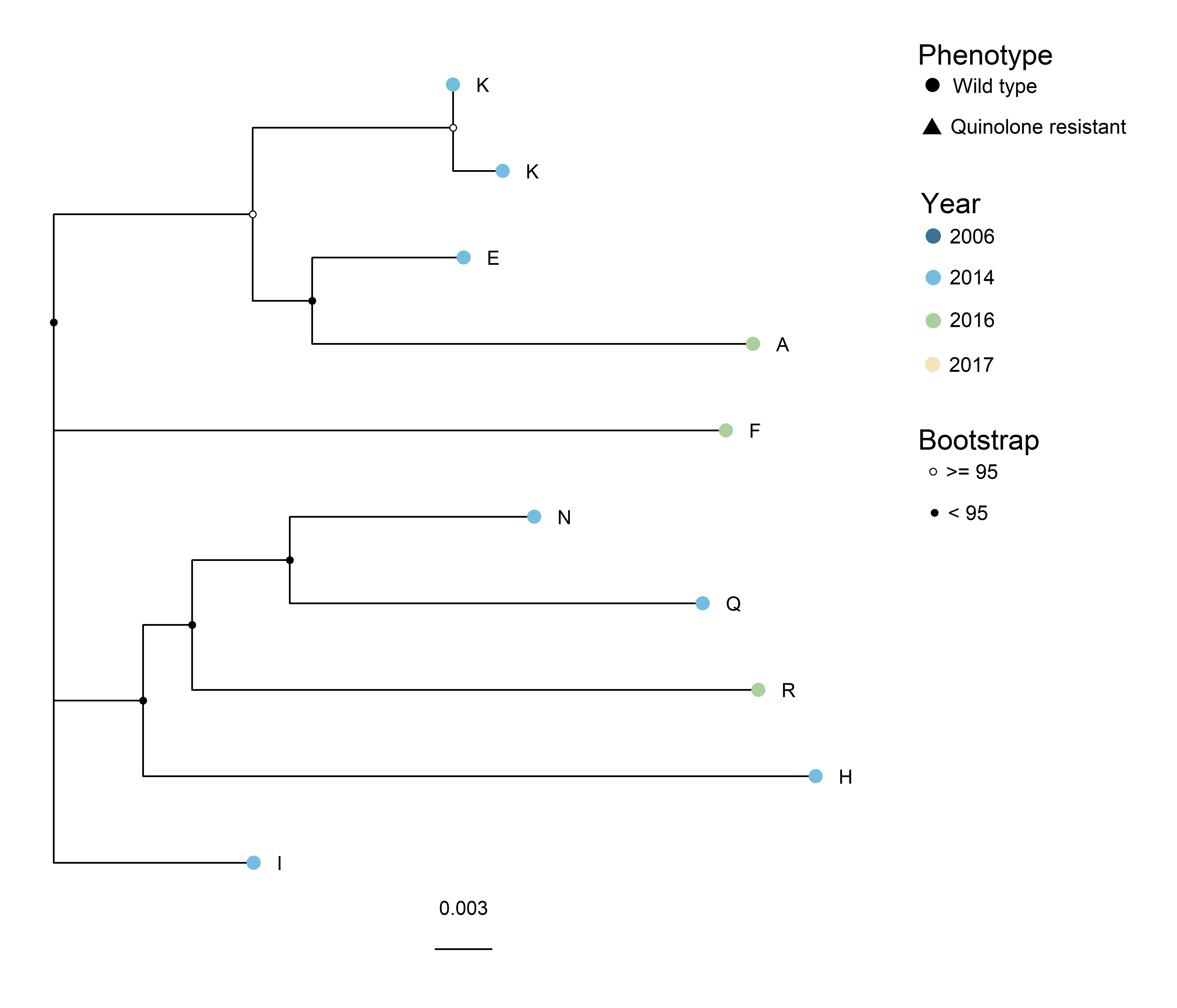


**Supplementary Figure 1:** Maximum likelihood core genome SNP tree generated with IQTree for clade B (ST5825). Core genome SNPs were identified with ParSNP, and recombinant sites were removed with Gubbins. Phenotype is represented by the tip point shapes, and year of isolation represented by the tip point colour. Bootstrap values are represented as black and white circles on the nodes. Median SNP distance for whole tree: 18 SNPs, with a range of 1 – 28 SNPs. Evolutionary model: K2P+ASC. Shared genome: 91.7%.


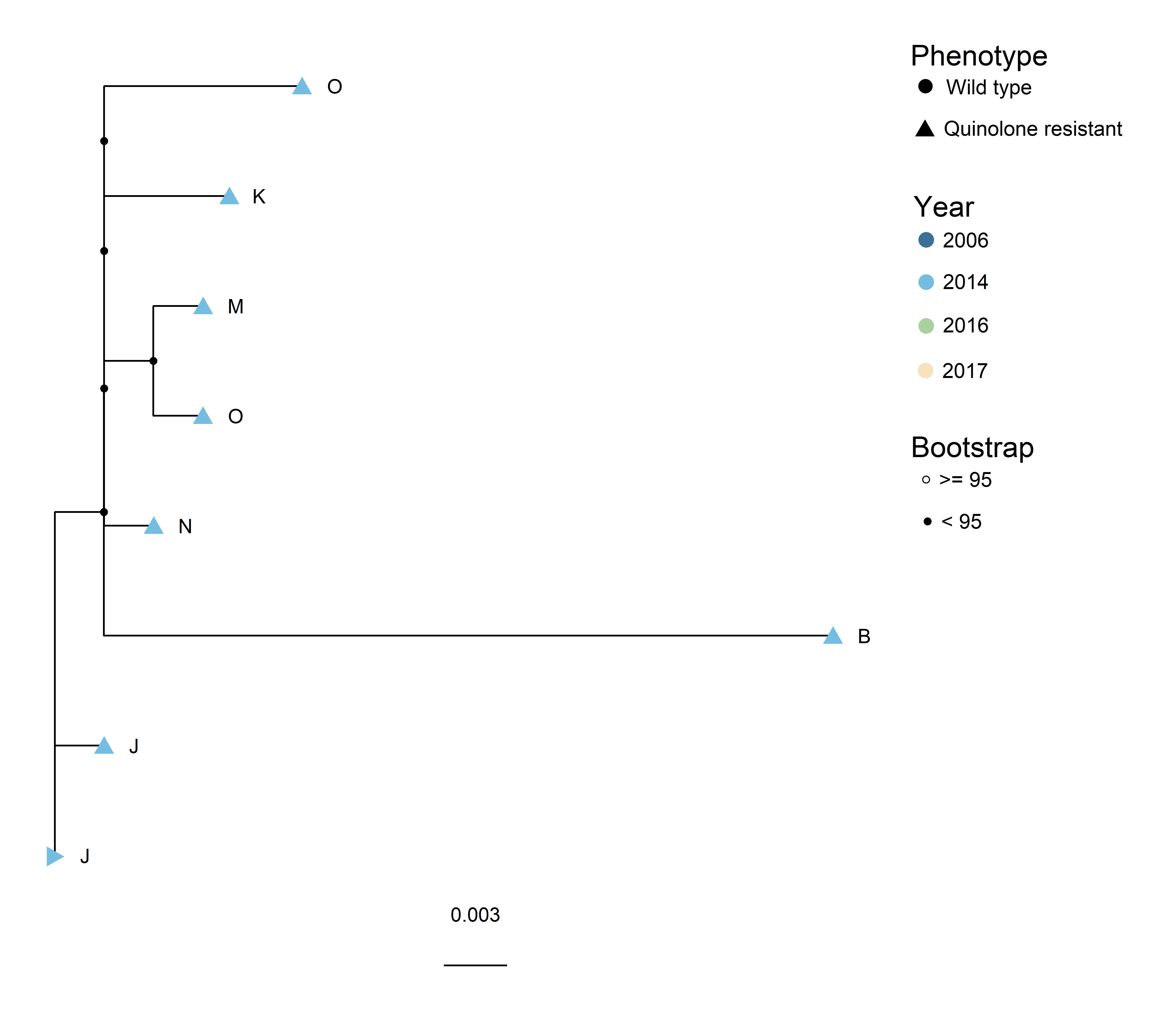


**Supplementary Figure 2:** Maximum likelihood core genome SNP tree generated with IQTree for clade D (ST349). Core genome SNPs were identified with ParSNP, and recombinant sites were removed with Gubbins. Phenotype is represented by the tip point shapes, and year of isolation represented by the tip point colour. Bootstrap values are represented as black and white circles on the nodes. Median SNP distance for whole tree: 9 SNPs, with a range of 2 – 37 SNPs. Evolutionary model: TIM3e+ASC. Shared genome: 92.4%.
